# Editorial: Media, Body Image, and Eating Behaviors

**DOI:** 10.3389/fpsyg.2022.851346

**Published:** 2022-04-07

**Authors:** Jinbo He, Shaojing Sun

**Affiliations:** ^1^School of Humanities and Social Science, The Chinese University of Hong Kong, Shenzhen, China; ^2^School of Journalism, Fudan University, Shanghai, China

**Keywords:** media, social media, body image, eating behaviors, disordered eating, body dissatisfaction

There has been ample evidence testifying to the connection between media, body image and eating behaviors (Holland and Tiggemann, 2016). For instance, past research has suggested that media exposure is linked to body and weight concerns (Perloff, 2014) and eating disorders (e.g., Anorexia Nervosa; White et al., 2016) directly or indirectly. Furthermore, current understanding of the tripartite relationship (media, body image, eating behaviors) largely rests with the era of mass communication. With sea changes taking place in the media landscape (e.g., diffusion of social media), the way people consume media information has been shifting and evolving. Media has become more diverse—including mass media, social media, intelligent media, among others—and its role in society is more powerful and penetrating. As such, there is an urgent need to revisit the relationship between media, body image, and eating behaviors in the contemporary media environment, leading to our conception of the present Research Topic of “*Media, Body Image, and Eating Behaviors*.”

The Research Topic opens with a systematic review by Zhang et al. Specifically, the systematic review extracted 87 effect sizes from 22 studies and revealed a pooled correlation of *r* = 0.09 (95% CI: 0.06, 0.11; *p* <0.001) between social network site (SNS) usage and disordered eating. Even though this is a small correlation, this review may provide further support for a connection between SNS usage and disordered eating. Moreover, Clark et al. contributed a review that summarizes the negative and positive influences of social media on weight stigma. They also provided insights and future research directions about how to address weight stigma on social media.

In this volume, two papers explored the effects of social media on body image and eating behaviors (Jackson et al.; Zhang et al.). More specifically, Jackson et al. investigated the knowledge, correlates, and effects of a social media meme of “the A4 challenge”, which involves demonstrating that one's waist can be completely hidden by a A4 sheet. The authors conducted three studies. In the first study, they found that compared to men, women were more likely to take the challenge. In the second study, they further explored whether there were differences in body image and disordered eating between women who passed the challenge and women who did not pass the challenge. Interestingly, they found that women who passed the challenge experienced fewer weight concerns and less appearance pressure than women who did not; however, there were no significant differences in certain disordered eating behaviors (e.g., binge eating). In the third study, the authors conducted an experiment to examine the effects of exposure in images of passing the challenge. However, the authors found that exposure to images of passing the challenge did not increase state body dissatisfaction, indicating that viewing images of passing the challenge might have no significant impact on state body dissatisfaction among women.

Zhang et al. experimentally examined the impact of high vs. low popular social media influencers' idealized body images on young Chinese females' body satisfaction and mood. Idealized body images refer to the images that shows the body representations that the viewers would be expected to aspire to. The study recruited female participants from a popular Chinese social media platform (RED) and set three experiment conditions (i.e., exposure to idealized body images from high-popular influencers, exposure to idealized body images from low-popular influencers, and a control condition of exposure to nature images). Results showed that exposure to idealized body images did not always produce harmful effects in young female social media users. Rather, for those with lower self-discrepancy between personal ideals and their own bodies, idealized body posts somewhat positively affected their body satisfaction.

To explore whether and how body dissatisfaction is related to impulse buying, Cai et al. conducted a mediation analysis with a sample of Chinese university students. Specifically, they observed that body dissatisfaction was positively associated with impulse buying among Chinese university students, and this association was further mediated by self-acceptance and self-esteem. Furthermore, the authors found that gender moderated the mediation model. These findings provide implications of reducing impulsive buying from the body image perspective.

Finally, the last article from Ouyang et al. explored the relationships between media internalized pressure, social physique anxiety, weight control self-efficacy and sports participation among Chinese university students. They found social physique anxiety and weight control self-efficacy played significant mediating roles in the relationship between media internalized pressure and sports participation; in other words, students who had higher levels of media internalized pressure tended to experience more social physique anxiety and, have higher weight control self-efficacy, which in turn predicted more sports participation. The findings of the study may indicate that the effects of media internalized pressure on wellbeing have two sides: on one hand, media internalized pressure has demonstrated to be a major source of body dissatisfaction (Knauss et al., 2008); on other hand, it may increase sports participation which improves wellbeing (Penedo and Dahn, 2005).

Overall, the collection of work encompasses various media platforms such mass media, SNS, and social media, and sheds light on the complex role and impact of media in the domain of body image and eating behaviors. However, compared to the wide range of media platforms, this collection is far from enough to fully uncover the linkage between different media, body image concerns, and eating behaviors. Future research should look into alternative media platforms, particularly those emerging and popular ones not examined here, such as Facebook, WeChat, and TikTok. Notably, this collection provides clinical implications for interventions and stimulates directions for future research aiming to further understand the topic of “*Media, Body Image, and Eating Behaviors*.”

## Author Contributions

Both authors co-wrote the editorial and approved the final version.

## Funding

JH received the Presidential Fund of The Chinese University of Hong Kong, Shenzhen (No. PF.01.001428) for the current editorial.

## Conflict of Interest

The authors declare that the research was conducted in the absence of any commercial or financial relationships that could be construed as a potential conflict of interest.

## Publisher's Note

All claims expressed in this article are solely those of the authors and do not necessarily represent those of their affiliated organizations, or those of the publisher, the editors and the reviewers. Any product that may be evaluated in this article, or claim that may be made by its manufacturer, is not guaranteed or endorsed by the publisher.
